# Anomalous Origin of Left Circumflex Artery from the Right Sinus of Valsalva in Cardiac Computed Tomography in a Group of 16,680 Patients—Radiologic and Clinical Characteristics

**DOI:** 10.3390/jcm12237240

**Published:** 2023-11-22

**Authors:** Alexander Suchodolski, Jan Głowacki, Mariola Szulik

**Affiliations:** 1 Doctoral School of the Medical University of Silesia, 40-055 Katowice, Poland; 2 Department of Cardiology, Congenital Heart Diseases and Electrotherapy, Silesian Center for Heart Diseases, Faculty of Medical Sciences in Zabrze, Medical University of Silesia, 40-055 Katowice, Poland; m.szulik@sccs.pl; 3 Department of Radiology and Radiodiagnostics, Faculty of Medical Sciences in Zabrze, Medical University of Silesia, 40-055 Katowice, Poland; j.glowacki@sccs.pl; 4 Computed Tomography Laboratory, Silesian Centre for Heart Diseases, 41-800 Zabrze, Poland; 5 Department of Medical and Health Sciences, Faculty of Applied Sciences, WSB University, 41-300 Dąbrowa Górnicza, Poland

**Keywords:** computed tomography, coronary anomaly, angiography, circumflex

## Abstract

Background: Anomalous aortic origin of a coronary artery (AAOCA) is the most prevalent form of coronary anomaly. One variant of AAOCA is the anomalous origin of the left circumflex artery from the right sinus of Valsalva, which can be detected using cardiac computed tomography (CT). However, limited data are available regarding the natural history of this anomaly, its impact on myocardial function, and associated symptoms. Methods: We conducted a retrospective analysis of 16,680 CT exams (cardiac and chest) performed between 2015 and 2022 at our Heart Imaging Department, utilising a dual-source 128-slice CT scanner (SOMATOM Definition Flash, Siemens Healthineers, Forchheim, Germany). A registry of patients with anomalous origin of the circumflex artery from the right sinus of Valsalva (RCx) was established. The study included 56 cases of RCx (0.33%). Clinical information was obtained from medical records. RCx was defined as a circumflex artery originating from the right sinus of Valsalva (type I or II) or the right coronary artery (type III). Two researchers independently reevaluated each CT exam in our study group to ensure accurate radiologic descriptions and provide additional precise radiologic information regarding the anomaly, including high-risk features. Results: Our study comprised 56 patients, with approximately equal distribution between males (*n* = 30, 54%) and females (*n* = 26, 46%), and with a median age of 59 years. Coronary heart disease (CAD) was known in 23% of patients (*n* = 13), while 11% (*n* = 6) were obese (defined as a BMI > 30 kg/m^2^), and 13% (*n* = 7) were diagnosed with type 2 diabetes. Only 9% of patients (*n* = 5) were smokers. Dyslipidemia was the most prevalent atherosclerotic risk factor, affecting approximately one third of patients (*n* = 17, 30%). In 14% (*n* = 8) of patients, heart failure was observed, while 13% (*n* = 7) were diagnosed with atrial fibrillation. Type I RCx was the most common subtype, identified in 48% of patients (*n* = 27) with this anomaly. Type II and Type III were found in 25% (*n* = 14) and 27% (*n* = 15) of patients, respectively. Conclusions: Our findings suggest that RCx is frequently encountered as an incidental finding, and we did not identify a consistent clinical characteristic in all patients with this type of anomaly. Furthermore, no gender predominance was associated with RCx. The natural history of this anomaly and its clinical implications seem benign. Further research is warranted to better understand this anomaly’s natural course and clinical implications.

## 1. Introduction

**Figure 1:**
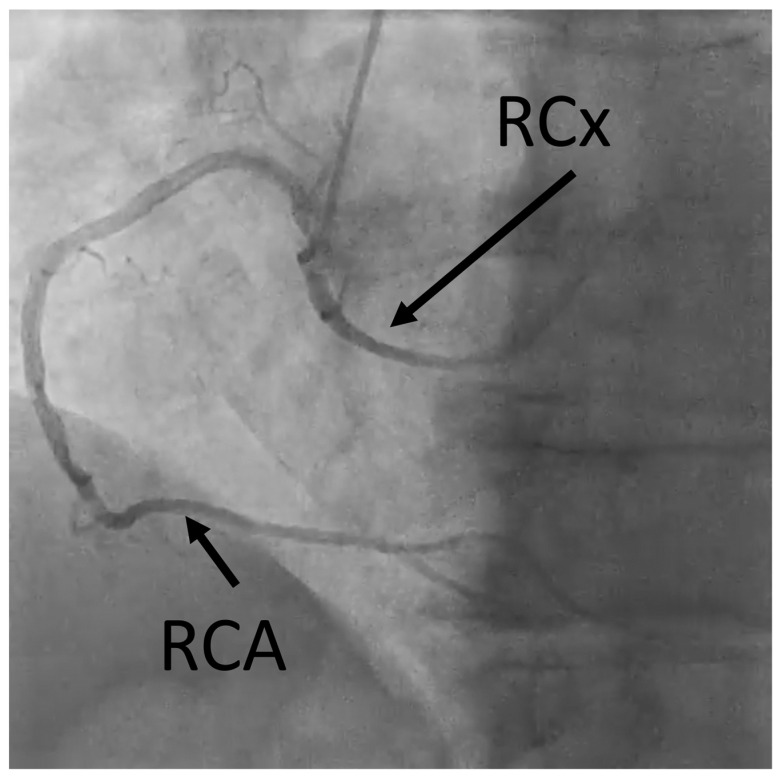
Coronary angiography image showing the right coronary artery (RCA) and anomalous origin of the left circumflex artery from the right sinus of Valsalva (RCx).

**Figure 2:**
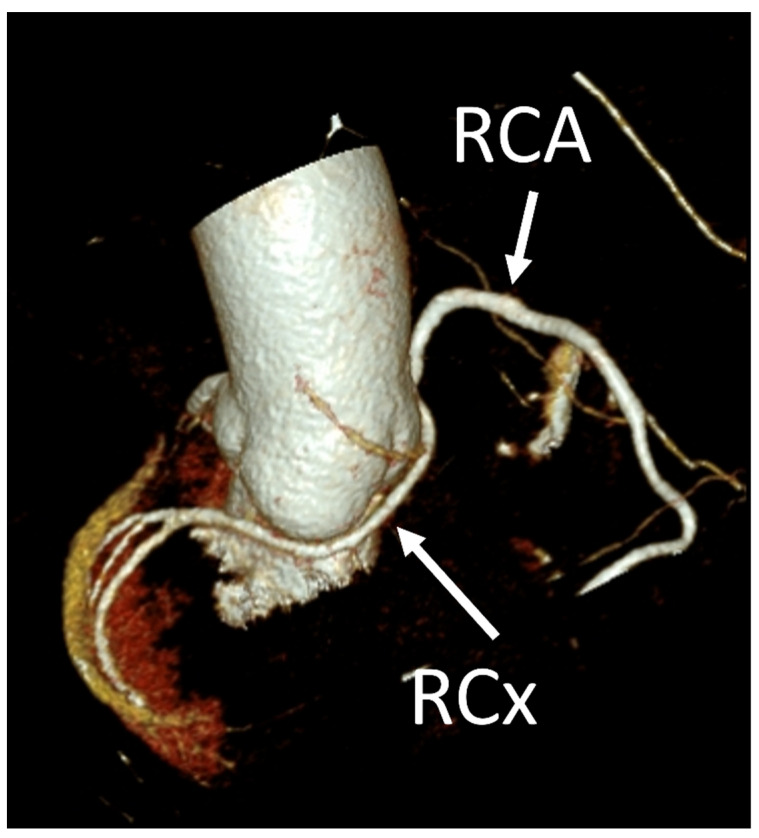
VRT (Volume Rendering Technique) CT image showing the anomalous origin of the left circumflex artery from the right sinus of Valsalva (RCx) in the same patient.

**Figure 3:**
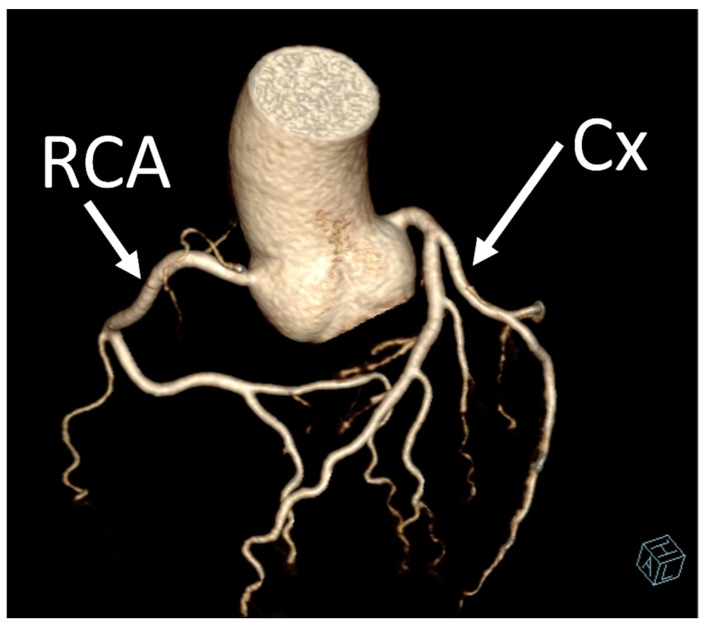
VRT (Volume Rendering Technique) CT image showing the normal origin of the left circumflex artery.

Coronary artery anomalies (CAAs) are rare congenital conditions with a variety of types and clinical symptoms. They are increasingly studied and recognized as more clinically important than they used to be, as cases of sudden cardiac death (SCD) have been linked to such anomalies [1]. Another factor contributing to this is that with wider usage of cardiac computed tomography angiography (CCTA) in everyday clinical practice, as recommended by European and American guidelines, more cases are detected [2,3]. The prevalence of CAAs varies heavily between studies, depending mostly on the method used to diagnose it. They occur in 0.6–2.1% of patients referred for invasive coronary angiography (ICA) [4,5]. (Figure 1) The prevalence seems to be much higher in studies using CCTA, where they are reported in 7.9–13.8% of results [4,5]. (Figure 2 and Figure 3) Noteworthy in a study by Adreini et al., 69.0% of them were diagnosed previously using ICA [4]. The vague definition of CAA can also explain these discrepancies. Some findings may be classified as one by some authors and as a variant of the norm by others. One example is myocardial bridging, which, due to it prevalence, is often not classified as an anomaly. Despite the rareness of this finding, coronary anomalies account for 33% of sudden non-traumatic deaths in young military recruits, as shown in an autopsy study from a 25-year-old period [6]. Anomalous aortic origin of a coronary artery (AAOCA) is a rare anomaly of coronary vasculature; however, it is the most common type among CAAs. According to a study using computed tomography by Krupiński et al., the prevalence of AAOCA was 0.76%. [7]. In another study by Opolski et al., a comparable result of 0.84% was found [8]. One of the variants of AAOCA is the anomalous origin of the left circumflex artery from the right sinus of Valsalva (RCx), which can be detected with cardiac computed tomography angiography (CCTA). This anomaly was first described by Antopol and Kugel in 1933. [9] The prevalence of anomalous circumflex arteries in the general population is estimated to be 0.02% to 0.6%. [10] Due to the rarity of this finding, there are limited data available on the natural history of this anomaly, its effect on symptoms, and myocardial function.

CCTA provides detailed information about the anatomy and course of the anomalous artery, which is crucial for proper diagnosis and treatment planning. Recent advances in CT technology and techniques have improved the accuracy and resolution of CT imaging, making it a valuable tool for identifying and characterizing this anomaly. In this article, we present the results of our study on the clinical, echocardiographic, and radiologic characteristics of patients with such an anomaly diagnosed via cardiac CT.

To our knowledge, this is the largest study to address this type of anomaly in such detail. Our findings may help understand this anomaly’s clinical significance and guide clinical decision-making for affected patients.

## 2. Methods

We evaluated 16680 CT exams (cardiac and chest) performed in our Heart Imaging Department between 2015 and 2022 on a dual-source 128-slice CT scanner (SOMATOM Definition Flash, Siemens Healthineers, Forchheim, Germany) and established a retrospective registry of patients with an anomalous origin of the circumflex artery from the right sinus of Valsalva (RCx). The studies were ordered according to current guidelines to evaluate patients with chest pain and to exclude clinically significant obstructive coronary artery disease. The study included 56 cases of RCx (0.33%).

Clinical information was obtained from the electronic medical record system of our institution with full anonymization of the study population.

Two researchers reevaluated each CT exam of our study group to check the radiologic description and add more precise radiologic information about the coronary anomaly, which may not have been routinely assessed.

We assessed the type of origin of RCx in each exam. Depending on the origin of the circumflex artery, we differentiated ones originating directly from the right sinus of Valsalva (type I or II) or from the right coronary artery (type III). Type I was characterized by a common origin of the right coronary artery and anomalous circumflex artery from the right coronary sinus, while in type II, these arteries had separate origins.

Retrospectively, the images were analyzed for high-risk anatomy features—as described in current medical literature. This included ostium morphology. Here, we differentiated between normal (round), oval, and slit-like ostia. The angle of anomalous artery take-off was measured and labelled as acute when the value was less than 45 degrees. If part of the vessel was visualized within the aortic wall, this was defined as an intramural course. In cases where, due to poor image quality, distinguishing between intramural and near-mural course was impossible, the anomalous coronary artery was also labelled as “intramural course”. Finally, we differentiated two courses of the proximal part of the artery: between the aorta and pulmonary trunk (so-called “malignant” course) and between the aorta and left atrium (“benign” course).

Echocardiographic data were obtained from the electronic medical record system of our institution. Information about valvular pathology and left atrial enlargement (defined as a diameter of >40 mm in the parasternal long axis view) were included. When there were no echocardiographic data for the patient, or the above-mentioned pieces of information were missing, the patient was excluded from this part of the study.

Statistical analyses were carried out using Excel 2016 and Statistica version 12.

## 3. Results

### 3.1. Clinical

Our study population consisted of 56 patients. About half of them were male (*n* = 30, 54%). Their age ranged between 15 and 84 years, with a median of 59 years. In about one in four patients (*n* = 13, 23%), coronary heart disease (CAD) had been known. They were diagnosed using CCTA according to current guidelines to exclude clinically significant obstructive coronary artery disease, so most likely, they had angina-like symptoms. However, in the available clinical records, this was not described precisely. In total, 11% (*n* = 6) of patients were obese (defined as a BMI > 30 kg/m^2^), and 13% (*n* = 7) were diagnosed with type 2 diabetes. Only five patients (9%) were smokers. The most common atherosclerotic risk factor was dyslipidemia—about every third patient (*n* = 17, 30%) had this diagnosis. Heart failure occurred in eight (14%) patients. Atrial fibrillation was diagnosed in seven patients (13%). All clinical characteristics are summarized in Table 1.

### 3.2. Radiologic

In our study, we reevaluated each CT exam to determine the type of anomalous origin of the circumflex artery. The most common type, Type I, which is defined as a common origin of the right coronary artery (RCA) and the circumflex artery (Cx) from the right sinus of Valsalva, was observed in 48% (*n* = 27) of patients. Type II, which is characterized by a separate origin of RCA and Cx from the right sinus of Valsalva, was identified in 25% of the patients (*n* = 14). In contrast, type III, where the Cx originates from RCA, was diagnosed in a similar number of patients (27%, *n* = 15) [11,12]. In Table 2, we show exemplary CT images of normal coronary vasculature, as well as the three types of RCx and diagrams explaining the correlation of coronary arteries in each. In Table 3, the results mentioned before are summarized.

To determine the potential for ischemia due to compression of the anomalous artery, we examined the course of the artery. All of the patients in our study had a “benign” course, meaning that the artery followed a course between the aorta and left atrium. Additionally, we evaluated other high-risk features of the anomalous coronary arteries. The shape of the RCx ostia was evaluated in each case. Round (or “normal”) is the most common, occurring in 75% (*n* = 42) of patients. An oval shape was found in 25% (*n* = 14) of cases, while slit-like ostia, considered the high-risk variant, were not observed in our study population.

Furthermore, we measured the take-off angle of the anomalous Cx and found that a majority of patients (70%, *n* = 39) presented an acute angle (defined as <45 degrees). These are also considered to be a high-risk feature in CAAs. In about half of the studies (52%), an intramural course of the artery was identified.

While type I was the most common type observed, all patients in our study presented a benign course of the anomalous artery. The findings regarding the shape and take-off angle of the RCx ostia may be helpful in planning surgical interventions for this anomaly. The detailed results are presented in Table 3.

### 3.3. Echocardiographic

Our study had a retrospective design; therefore, not all patients had complete echocardiographic data available for analysis. Among the patients included in our study, we were able to obtain the complete basic echocardiographic data for 37 patients. Mitral regurgitation was the most commonly observed valve pathology, which was present in 38% (*n* = 14) of cases. Most of them were mild, and only one was moderate. Mitral stenosis was not detected in any of the patients included in our study. Aortic valve pathologies, including stenosis and regurgitation, were observed in 8% (*n* = 3) of the cases.

The second most frequently observed lesion was tricuspid regurgitation, identified in 22% (*n* = 8) of the patients. Most of them were mild, and only one was severe. However, we did not find any cases of tricuspid stenosis. In addition, we also analyzed left atrial size and found left atrial enlargement, defined as a diameter greater than 40 mm in the parasternal long-axis view, in 30% (*n* = 11) of the patients.

Overall, our findings suggest that mitral regurgitation is the most common valve pathology in the patients included in our study, while mitral stenosis was not observed. Aortic valve pathologies were uncommon, and tricuspid regurgitation was the second most frequently observed lesion. Additionally, we found left atrial enlargement in a significant proportion of our study population. The detailed echocardiographic findings are presented in Table 4.

## 4. Discussion

**Figure 4:**
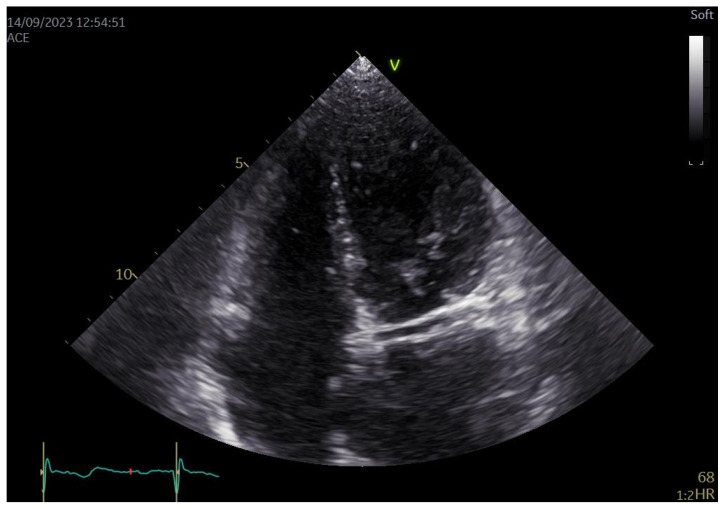
Transthoracic echocardiographic image of RAC (retroaortic anomalous coronary) sign in a patient with anomalous origin of the left circumflex artery from the right sinus of Valsalva (RCx). Apical 4 chamber view.

Anomalies of the coronary arteries are infrequent and, therefore, not widely studied. Among 16,680 studied patients, only 56 had this anomaly (0.33%). This prevalence corresponds with the one reported in other cohort studies [13]. The clinical impact of such coronary anomalies is yet to be determined. RCx is usually described in the literature in the form of case reports due to its apparent rareness. In this original article, we tried to show that this kind of anomaly, with a prevalence of 0.33%, will most likely be encountered by clinicians and radiologists specializing in the field of cardiovascular diseases. In our experience, RCx is most often an incidental finding. While it may be identified using ICA, in the experience of the authors, CCTA is the method of choice in the assessment of coronary anomalies. It can be challenging to recognize it using ICA (where a total obstruction of Cx may be diagnosed), and CCTA allows for three-dimensional imaging, allowing to establish the course of the vessel, which is crucial in the case of RCx and CAAs in general, as mentioned in the previous part of this paper [12]. Initial diagnosis can also be made using echocardiographic methods. In a recent paper, transthoracic echocardiography (TTE) showed a high (99.8%) diagnostic accuracy in RCx using “double binary tubular image” (DBTI) [14]. This echocardiographic sign is more widely described as an RAC (retroaortic anomalous coronary) sign. Another sign of a retroaortic anomalous coronary course is the bleb sign. Other authors also report the high specificity and lower sensitivity of such findings [15,16]. In the experience of the authors, this sign has to be used with caution, as the coronary sinus may mimic it in some patients. In Figure 2, we show an exemplary echocardiographic image of RAC sign in a patient with RCx. (Figure 4) We have to remember that they still have to be evaluated using the above-mentioned methods (ICA and CCTA) to confirm the diagnosis and make a more precise assessment, as explained earlier. There seems to be no gender predominance. We have not found a clinical characteristic that occurs in all patients with this kind of coronary anomaly.

Video S1 Coronary angiography shows the right coronary artery (RCA) and anomalous origin of the left circumflex artery from the right sinus of Valsalva (RCx).

Atrial fibrillation (AF) has been diagnosed in 13% of patients with RCx. As the left atrium is vascularized by the circumflex artery, potential ischemia of this coronary branch may lead to an increased likelihood of this arrhythmia [17]. In comparison, in a general population-based study by Davis et al., AF was diagnosed in 1.6% of women and 2.4% of men over the age of 45 years [18]. Therefore, the prevalence among patients with RCx seems much higher. However, in the NOMAD-AF study, a nationally representative random sample of Polish citizens over 65 years old, AF was diagnosed in 19.2% of cases [19]. As this was also a Polish registry with a median age of 59 years, the findings seem more comparable to the results of Kalarus et al. A potential influence of this coronary artery on the occurrence of AF seems unlikely. Nonetheless, studies specifically designed to answer this question are needed for definitive statements.

The most common type of RCx in our study (Type I, common origin of the RCA and Cx from the right sinus of Valsalva) was observed in almost half of the patient group. In another study, type II (separate ostia) was found in 36% of cases, compared to 25% in our group [20]. The currently available literature shows that specific radiologic features of anomalous coronary arteries, especially the course, may impact patients’ outcomes. Using an experimental animal model, Bartoli et al. showed that this kind of artery compression between the great vessels causes regional ischemia [21]. None of the circumflex arteries had this so-called malignant course in our cohort. Other unfavorable features include an acute angle takeoff (which in our group has been found in 70% of cases), intramural course (found in 52% of cases), and slit-like ostium (which did not occur in our study population) [1]. A recent study by Ratti et al., in which 14 athletes with RCx were described, did not include a case of malignant course and slit-like ostium. However, one of the high-risk features, namely acute angle take-off, was also found. In their population, it occurred much less often, in 21% of cases. Intramural segments have not been described by these authors. Differences in the studied groups or different radiologic assessments may explain this. Arteries with a close mural course may be described as ones with intramural course—distinguishing this feature is challenging, especially in low-quality images [20]. A case report by Rozenman et al. of a 75-year-old patient presenting with angina induced by exercise showed that an acute angle take-off in the absence of a malignant artery course and CAD could cause ischemia confirmed by single photon emission computed tomography (SPECT) [22]. This finding has been reported in other case reports since. Del Torto et al. described the case of a 46-year-old man with a benign RCx course, no CAD, and acute take-off, who had a myocardial infarction of the wall region supplied by the circumflex artery, confirmed using cardiac magnetic resonance (CMR) with late gadolinium enhancement (LGE) [23]. An explanation of this, proposed by Mahowald et al., is that the anomalous origin creates a flap-like mechanism during cardiac motion [24]. The available literature suggests that not only the vessel course but other high-risk features have to be taken into clinical consideration by clinicians taking care of such patients. These anatomical features may be of interest to radiologic or interventional diagnosticians; as mentioned before, this is one of the most commonly occurring coronary anomalies, so knowledge about the most probable phenotype helps in the assessment. Another topic regarding these vessels is the incidence of atherosclerosis. While some authors suggest an increased risk of this pathologic process in anomalous coronary arteries like RCx, a study by Mahson et al. using invasive coronary angiography (ICA) showed no increased incidence of atherosclerosis [25,26]. This seems to be in alignment with our experience. In a case report with autopsy, histology confirmed the absence of obstructive atherosclerotic coronary artery disease in RCx [27]. In our study, the small sample and limited retrospective data make assumptions about risk assessment or causality concerning the potential effect of RCx on myocardial function impossible. Prospective studies are needed to answer these kinds of questions.

Mitral regurgitation (MR) was the most prevalent valvular pathology in our study population, occurring in 38% of cases. Most of them were mild, and only one was moderate. For comparison, in the Framingham Heart Study, MR of more than or equal to mild severity was found in 19.0% of men and 19.1% of women, respectively [28]. The lack of more severe forms of this lesion in a congenital condition, affecting individuals since birth, makes a potential underlying ischemic mechanism unlikely. Furthermore, the results may be influenced by the fact that most patients were middle-aged and had cardiovascular comorbidities. However, further echocardiographic studies with proper control groups and more detailed evaluation of the heart valves are needed to answer such questions.

## 5. Conclusions

Based on our experience, it is common to come across RCx as an incidental finding, and we have not been able to identify a consistent clinical characteristic that is present in all patients with this type of coronary anomaly. There is no gender predominance associated with RCx.

It seems to be a benign anomaly with little impact on the clinical course of patients with it.

RCx and other CAAs are findings that interventional cardiologists should consider, as they may mimic obstructive coronary artery disease if not assessed properly during invasive coronary angiography. Knowledge about the prevalence of different origin types, as presented in this paper, should be helpful in this matter.

When it comes to assessing this type of anomaly, we have found that the method of choice is CCTA (Coronary Computed Tomography Angiography), as it provides a three-dimensional visualization. This type of imaging may be particularly beneficial when planning surgical procedures, such as coronary artery bypass grafting (CABG), when surgery is indicated because of other reasons. As we showed in this paper, isolated RCx is not an indication of surgery as the clinical course is benign in most cases. Moreover, CCTA is a non-invasive technique and, therefore, may be used in a broader patient population, which can help to reduce the need for more invasive diagnostic procedures like coronary angiography and minimize the risk of complications.

While there is still much to be learned about RCx anomalies, utilizing CCTA imaging can help with proper diagnosis and planning for medical interventions, if necessary.

## 6. Limitations

One of the major limitations of this study is the lack of echocardiographic data on all patients with this anomaly. A prospective study assessing more detailed echocardiographic parameters of this group of patients would offer more insight into the pathophysiology of this coronary anomaly—especially using speckle tracking echocardiography, which is proven to be an early marker of the deterioration of systolic function. This technique also allows for a precise measurement of wall motion abnormalities, a crucial parameter in the assessment of CAD [29]. Further research using this technique would add valuable information to the scarce knowledge about CAAs in general.

Another limitation of this retrospective cohort study is the lack of more extensive clinical data on each patient included in the analysis, especially more information about symptoms with an emphasis on stenocardia, arrhythmias, and decreases in exercise tolerance.

## Figures and Tables

**Table 1:** Clinical characteristics.

Characteristics	*n* = 56	100%
Male	30	54
Female	26	46
Heart failure	8	14
CAD	13	23
AF	7	13
DM t. 2	7	13
Obesity	6	11
Dyslipidemia	17	30
Smoking	5	9

CAD—coronary heart disease, AF—atrial fibrillation, DM t. 2—type 2 diabetes mellitus.

**Table 2:** RCx types with exemplary images.

Type	Diagram	CT Image
Normal	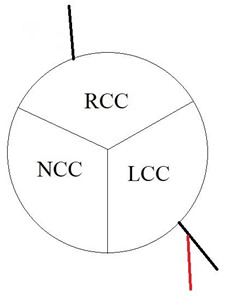	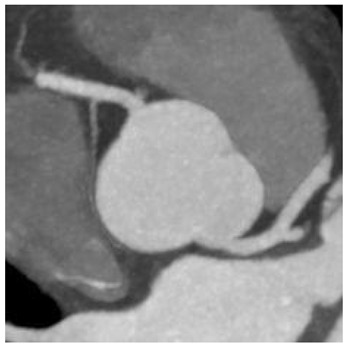
I	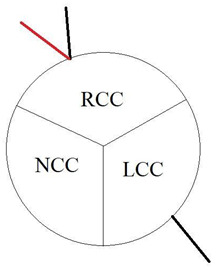	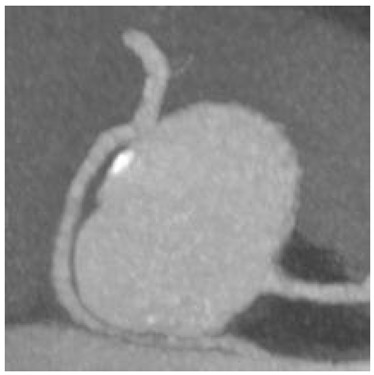
II	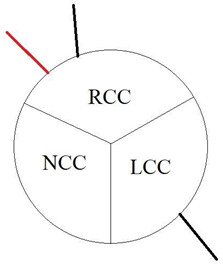	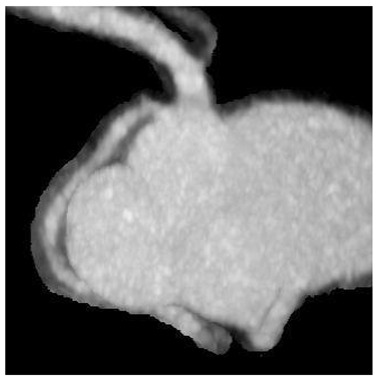
III	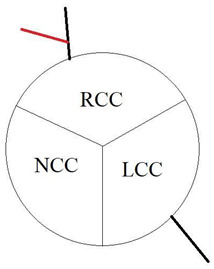	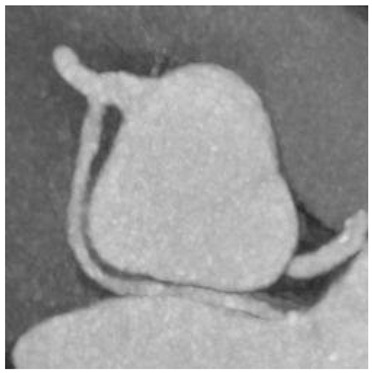

RCC—right coronary cusp, LCC—left coronary cusp, NCC—noncoronary cusp.

**Table 3:** Radiologic features of RCx.

Radiologic Feature	Types	Number of Patients	%
Origin	I	27	48
	II	14	25
	III	15	27
Course	Between aorta and pulmonary trunk “malignant”	0	0
	Between aorta and left atrium “benign”	56	100
Ostia	Round (normal)	42	75
	Oval	14	25
	Slit	0	0
Angle	≤45	39	70
	>45	17	30
Intramural course		29	52

**Table 4:** Echocardiographic evaluation (*n* = 37).

Echocardiographic Feature	Number of Patients	%
Enlarged LA	11	30
MR	14	38
MS	0	0
AR	3	8
AS	3	8
TR	8	22
TS	0	0

LA—left atrium, MR—mitral regurgitation, MS—mitral stenosis, AR—aortic regurgitation, AS—aortic stenosis, TR—tricuspid regurgitation, TS—tricuspid stenosis.

## Data Availability

Data is contained within the article and Supplementary Materials.

## Supplementary Materials

The following supporting information can be downloaded at: https://www.mdpi.com/article/10.3390/jcm12237240/s1, Video S1: Coronary angiography of anomalous origin of the left circumflex artery from the right sinus of Valsalva.
